# Six-minute walk distance in healthy subjects: reference standards from a general population sample

**DOI:** 10.1186/s12931-022-02003-y

**Published:** 2022-04-05

**Authors:** Lucia Cazzoletti, Maria Elisabetta Zanolin, Gianluigi Dorelli, Pietro Ferrari, Luca Giuseppe Dalle Carbonare, Ernesto Crisafulli, Mulubirhan Assefa Alemayohu, Mario Olivieri, Giuseppe Verlato, Marcello Ferrari

**Affiliations:** 1 Unit of Epidemiology & Medical Statistics, Department of Diagnostics and Public Health, University of Verona, Strada Le Grazie, 8, 37134 Verona, Italy; 2 School of Medicine in Sports and Exercise, Department of Medicine, University of Verona, Verona, Italy; 3 Department of Medicine, Respiratory Medicine Unit, University of Verona and Azienda Ospedaliera Universitaria Integrata of Verona, Verona, Italy; 4 Department of Epidemiology, School of Public Health, Mekelle University, Mekelle, Tigray Ethiopia; 5 Unit of Occupational Medicine, Department of Diagnostics and Public Health, University of Verona, Verona, Italy

**Keywords:** Reference values, 6-min walking test, Function status (activity levels), General population, Healthy subjects

## Abstract

**Introduction:**

The 6-min walking distance (6MWD) test is a useful tool to obtain a measure of functional exercise capacity. However, reference equations have been mainly based on selected populations or small samples. The purpose of this study was to determine the reference equations to predict the 6MWD in a large Italian population sample of healthy adults of a wide age range.

**Methods:**

In the frame of the multi case–control population-based study Gene Environment Interaction in Respiratory Diseases (GEIRD), we studied 530 healthy subjects: 287 females ranging 21–76 and 243 males ranging 21–78 years of age. We measured 6MWD, demographic and anthropometric data and collected the reported physical activity. A multiple linear regression model for the 6MWD included age, age^2^, height, weight and physical activity for both sex equations. The two-way interaction age-height and age-weight and the quadratic terms of weight and height were also tested for inclusion separately in each model.

**Results:**

The mean ± SD for 6MWD was 581.4 ± 66.5 m (range 383–800 m) for females and 608.7 ± 80.1 m (range 410–875 m) for males. The reference equations were 6MWD = 8.10*age + 1.61*height_cm_−0.99*weight_kg_ + 22.58*active−0.10*age^2^ + 222.55 for females (R squared = 0.238) and 6MWD = 26.80*age + 8.46*height_cm_−0.45*weight_kg_−2.54*active−0.06*age^2^−0.13*age*height_cm_−890.18 for males (R squared = 0.159), where “active” is 1 when the subject is physically active, 0 otherwise.

**Conclusion:**

This study is the first to describe the 6MWD in a large population sample of young, middle aged and elderly healthy Caucasian subjects, and to determine reference equations. These findings will help to improve the evaluation of Italian and European patients with diseases influencing their functional capacity.

## Introduction

The ability of walking is a well-established index of the functional capacity and the quality of life.

The 6-min walk distance (6MWD) test is a quick, easy and inexpensive tool for measuring functional exercise capacity and it is very useful in elderly, frail patients or those with chronic diseases who are unable to perform maximal cycle ergometer or treadmill exercise tests.

The 6MWD test has advantages over laboratory-based tests on exercise tolerance since it more closely reflects the ability to perform daily life activities and does not require sophisticated and expensive equipment [1–3]⁠. Moreover, the test is frequently employed as an outcome measure for pulmonary and cardiovascular rehabilitation, and as a selection tool for pulmonary surgery candidates [4, 5]⁠.

Most of the studies on 6MWD were conducted in groups of patients with physical function alterations, such as obstructive lung disease, heart failure, neuromuscular diseases and arthritis [1, 2]⁠. Several studies have been conducted in healthy adults over the last decades to provide reference values for the 6MWD. The majority of these were performed on a low number of subjects (i.e. < 100) [6–9], and/or in samples of a narrow age-range [6, 9]. Furthermore, participants were generally recruited among students, employees and relatives [10–13], while only one study considered a sample from the general population [14]. Some studies included patients with asthma, a disease associated with a reduction in the 6MWD, independently of normal respiratory function, in the group of healthy subjects [15]. All these concerns may lead to biases when formulating reference values for use in clinical practice.

The aim of this study was to identify the reference equations for predicting 6MWD for women and men in a large Italian population sample of healthy adults of a wide age range, thereby contributing to improve the evaluation of European patients with diseases affecting their functional capacity.

## Methods

### Study population

The subjects participated in the Genes Environment Interaction in Respiratory Diseases (GEIRD) project, a two-stage multicase-control study, carried out in Verona (Italy) between 2008 and 2012. (www.geird.org) [16].

Briefly, in stage 1, new random samples or pre-existing randomly sampled cohorts from the general population (20–85 years of age, male/female = 1/1) were mailed a screening questionnaire on respiratory symptoms [17]. In stage 2, all the subjects who reported symptoms suggestive of asthma or chronic bronchitis (CB), a random sample (30%) of the subjects who reported rhinitis and a random sample (40%) of the subjects who did not report respiratory symptoms, diagnoses or hospitalizations were invited to clinics, where they underwent interviews and clinical tests.

Ethical approval was obtained from the appropriate ethics committee, and written consent was obtained from each participant.

In our analysis, only the subjects eligible for the 6-min walking test (6MWT) protocol were considered. In particular, a screening questionnaire was mailed to 8206 subjects, and 5647 (response rate: 69%) answered; out of 3552 selected to participate in the clinical stage, 1453 (41%) accepted to undergo clinical interviews and tests and were screened for inclusion in the healthy group of subjects considered in this study.

### Procedures

Data on smoking, respiratory or other diseases, use of medications were collected through a standardized questionnaire [16]. The subject’s standing height was measured by using a stadiometer; weight was measured by a balance beam scale. The subjects underwent lung function tests according to ATS/ERS guidelines [18], with reference values obtained by Quanjer [19].

The subjects were classified on the basis of physical activity. We followed the definition used by Shabaan et al. [20], who based their categorisation on earlier publications [21, 22]: subjects who performed physical activity with a frequency of ‘‘2–3 times a week’’ or more and with a duration of ‘‘about 1 h a week’’ or more were classified as ‘‘active’’, the remaining subjects as ‘‘not active’’.

The 6MWT protocol was developed according to the American Thoracic Society guidelines (2002) [23]. Subjects were checked for contraindications, then asked to walk as far as they could without running in a 30-m-long hallway. If a subject stopped before the 6 min were up (or the operator decided that he/she should not continue), the reason for stopping was recorded. The test results were expressed as the distance walked (i.e. the 6MWD) in meters.

### Definition of the healthy group and statistical method

We excluded cases of asthma, COPD, CB according to our previous analysis showing a significant reduction of the 6MWD in these subjects [15]. Out of the remaining 816 subjects, we also excluded 102 subjects who did not perform the test, due to clinical contraindications, such as heart attack occurred in the previous 3 months, current drug treatment for epilepsy, a resting heart rate greater than 120 beats per minute, a baseline systolic blood pressure greater than 180 mmHg or a diastolic blood pressure greater than 100 mmHg. Finally, we excluded 7 subjects with values of FEV1 lower than normal according to the z-score calculated by means of the global lung function 2012 equations [19], 2 subjects with 6MWD >  = 900 m (a distance reached only by running), and 4 subjects with no information on physical activity. Thus, we considered 701 subjects for the subsequent analysis aimed to define the healthy group. For this purpose, we excluded the subjects with features other than respiratory diseases that were negatively associated with the 6MWD [14]. If the variable was continuous, participants beyond the 95th percentile toward abnormality were excluded. Multivariable linear regressions were then fitted to data to detect disease conditions negatively associated with the 6MWD. Each regression considered the 6MWD as dependent variable, and age, sex, smoking habits, BMI and one at a time other potentially relevant factors (such as reported cardiovascular diseases, arthritis, diabetes, cancer, hypertension and obstructive sleep apnoea syndrome) as independent variables.

When the sample of subjects meeting the inclusion criteria to be defined as healthy was obtained, two multivariable linear models were fitted to data. The 6MWD was considered as the dependent variable and age, age squared, height, weight and physical activity were selected as independent variables, in males and females separately. In addition, the two-way interaction terms of age and height and age and weight were tested by the likelihood ratio test (LRT), one at a time. Finally, height squared and weight squared were included in the models one at a time, if the Ramsey regression specification-error test for omitted variables was significant [24]. The final models were tested for heteroscedasticity [25], and Huber–White standard errors were used to take heteroscedasticity into account [26]. Age, height and weight were centred separately in males and females.

The reference equations for females and males were calculated considering that the estimates of the coefficients have been obtained with centred variables.

We calculated the 5th percentile lower limit of normal (LLN) using the estimated reference equations for 6MWD; the calculated LLN is:

$${\text{Predicted mean }}{-}{ 1}.{645 }*{\text{ RSD}}$$

where RSD is the residual standard deviation of the estimated regression [27].

## Results

Out of the 701 subjects who completed the 6MWT, 171 were excluded as they exhibited disease-related features negatively associated with 6MWD. In particular, the initial regression models detected the factors associated with a reduced 6MWD: current smoking and diabetes. Moreover, BMI was negatively associated with 6MWD. Out of the 171 subjects, 133 (77.7%) were current smokers, 17 (9.9%) reported diabetes and 35 (20.5%) had a BMI value beyond the 95th percentile of the whole group (Table 1). Reported cardiovascular diseases, arthritis, cancer, hypertension, obstructive sleep apnoea syndrome and former smoking were not associated with the 6MWD.

**Table 1:** Number of subjects (% out of the number of excluded subjects: 171) excluded from the healthy group for each variable found to be negatively associated with the 6MWD

	n (%)
Current smoking	133 (77.7)
Reported diabetes	17 (9.9)
BMI > 95th percentile	35 (20.5)

One hundred and seventy-one participants were excluded; the difference with the 185 given here is because some participants had more than one factor that excluded them from the healthy subset

The remaining 530 subjects were considered to determine the reference equations of 6MWD. Table 2 shows their characteristics. The participants were distributed by age range as follows: 155 (29.3%) were young (< 39 yrs), 332 (62.6%) were middle-aged (40–64 yrs) and 43 (8.1%) were elderly (65–78 yrs).

**Table 2:** Main characteristics of female and male subjects used to determine reference values of 6MWD (n = 530)

	Females n = 287	Males n = 243
Age, years		
Mean (s.d.)	46.4 (10.9)	47.1 (12.8)
Range	21.2–75.8	21.2–77.8
Height, cm		
Mean (s.d.)	163.0 (6.5)	175.9 (6.7)
Range	146.0–186.0	156.0–192.0
Weight, Kg		
Mean (s.d.)	61.7 (9.5)	78.6 (9.9)
Range	41.0–106.0	57.0–107.0
BMI		
Mean (s.d.)	23.2 (3.4)	25.4 (2.8)
Range	16.9–31.2	17.6–31.6
Smoking habits, n (%)		
Non smokers	216 (75.5)	137 (56.4)
Ex-smoker	70 (24.5)	106 (43.6)
Physically active, n (%)	119 (41.5)	112 (46.1)
6MWD, m		
Mean (s.d.)	581.4 (66.5)	608.7 (80.1)
Range	383.0–800.0	410.0–875.0
FEV1% predicted, %		
Mean (s.d.)	103.7 (12.4)	104.5 (11.4)
Range	77.3–166.9	80.6–159.9
FEV1/FVC, %		
Mean (s.d.)	84.2 (6.1)	82.7 (6.6)
Range	70.1–103.7	66.9–103.8

The linear regression model fitted to data for females (n = 287) is presented in Table 3. The interaction terms of age and height and of age and weight were not included in the current model (LRT: p = 0.953 and p = 0.873, respectively). The model did not present evidence of omitted variables (p = 0.179) and the test for heteroscedasticity was not statistically significant (p = 0.125).

**Table 3:** Regression coefficients, 95%CI and p-value of the Linear Regression Model for the outcome 6MWD – Females

	β	95% CI	P
Age (years)	− 1.24	(− 1.90, − 0.58)	< 0.001
Height (cm)	1.61	(0.35, 2.86)	0.012
Weight (kg)	− 0.99	(− 1.70, − 0.28)	0.006
Active *vs* not active	22.58	(8.86,36.30)	0.001
Age^2^ (years^2^)	− 0.10	(− 0.14, − 0.05)	< 0.001
Constant term	583.48	(573.43, 593.54)	< 0.001

R squared = 0.2381. The regression model was estimated using centered variables

The linear regression model fitted to data for males (n = 243) is presented in Table 4. Differently from women, the interaction term of age and height was relevant in the male model (LRT: p = 0.025), while the interaction of age and weight was excluded (LRT: p = 0.710). The model with the interaction between age and height did not present any evidence neither of incorrect specification (p = 0.960) nor of heteroscedasticity (p = 0.809).

**Table 4:** Regression coefficients, 95%CI and p-value of the Linear Regression Model for the outcome 6MWD – Males

	β	95%CI	p
Age (years)	− 1.72	(− 2.60; − 0.85)	< 0.001
Height (cm)	2.34	(0.64; 4.04)	0.007
Weight (kg)	− 0.45	(− 1.67; 0.77)	0.466
Active *vs* not active	− 2.54	(− 21.75; 16.66)	0.794
Age^2^ (years^2^)	− 0.06	(− 0.11; 0.00)	0.042
Age*Height (years*cm)	− 0.13	(− 0.23; − 0.03)	0.012
Constant term	615.18	(598.30; 632.07)	< 0.001

(R squared = 0.1592). The regression model was estimated using centered variables

**Fig. 1:**
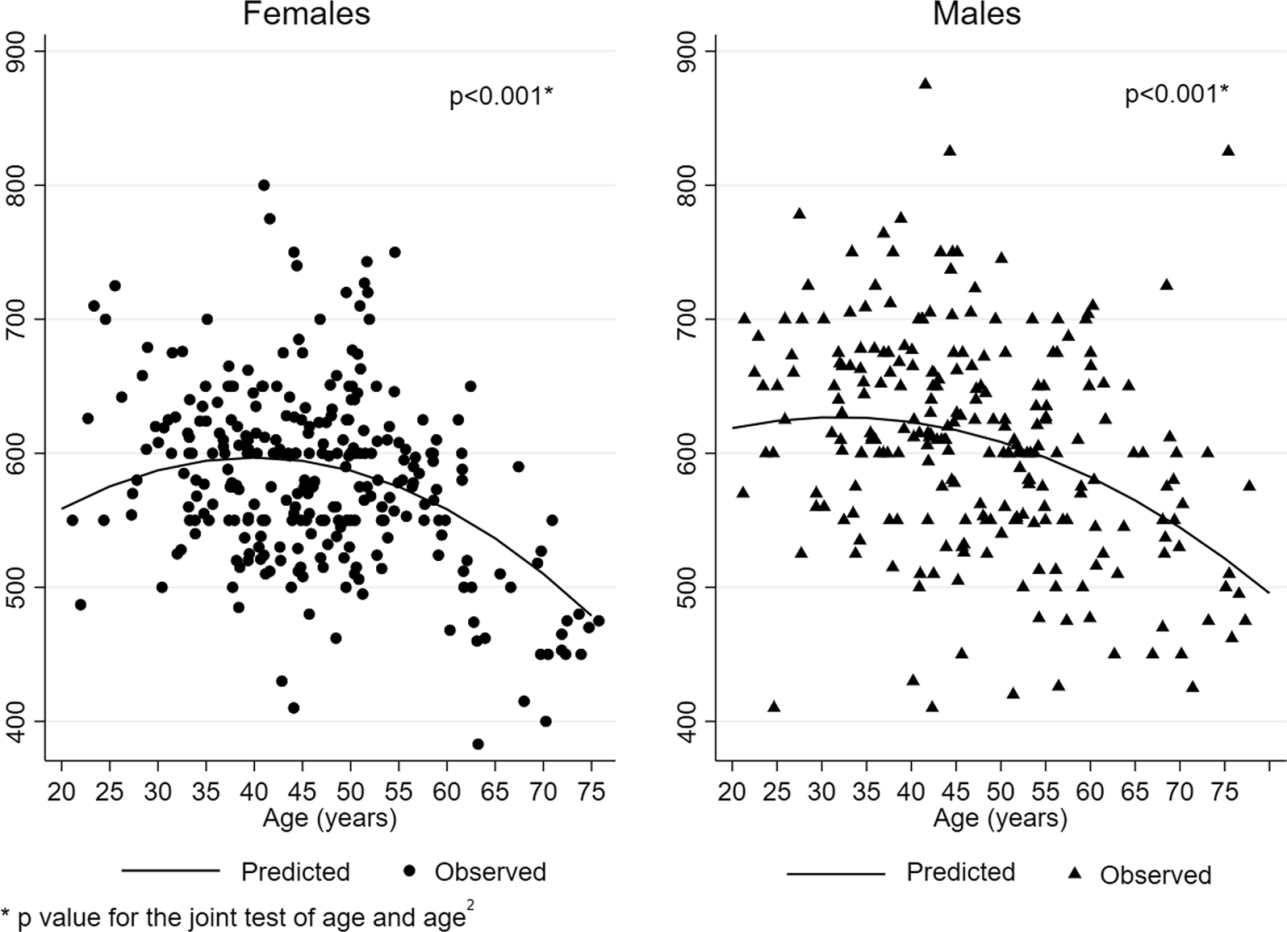
Observed (circle and triangles markers) and adjusted marginal mean (line) 6MWD by age in females and in males

**Fig. 2:**
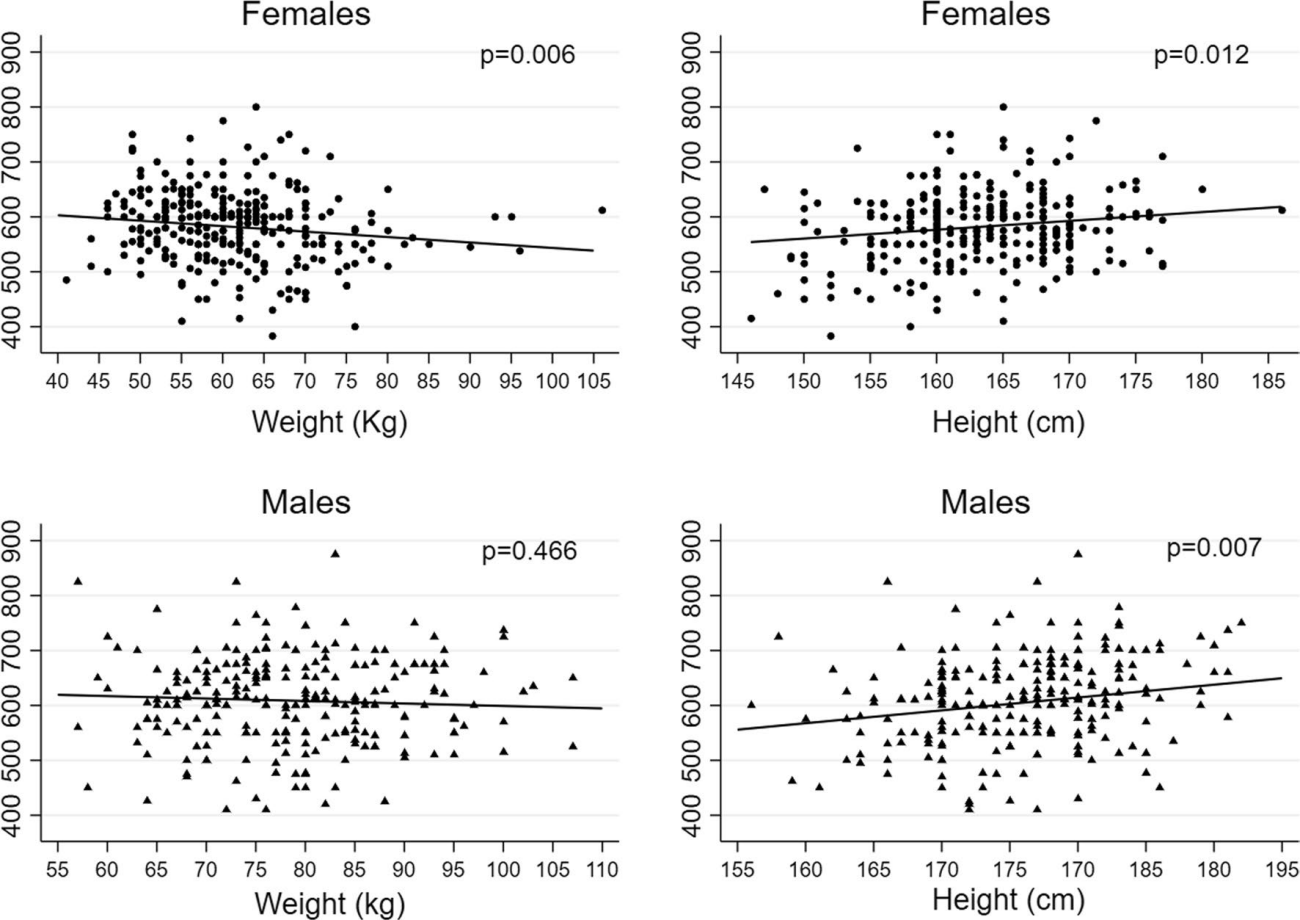
Observed (circle and triangles markers) and adjusted marginal mean (line) 6MWD by weight and height in females and in males

**Fig. 3:**
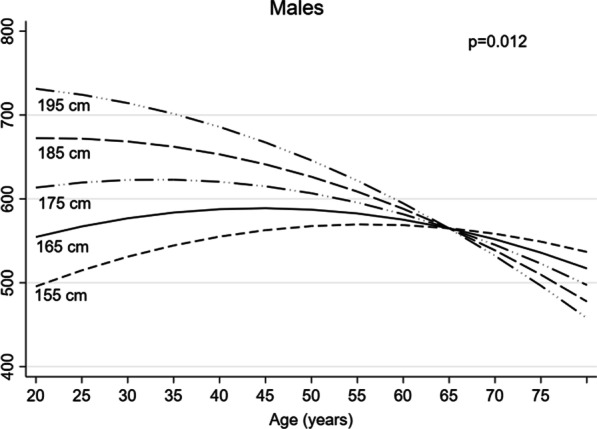
Adjusted marginal mean 6MWD by age and height in males

Figure 1 shows the observed and adjusted marginal mean 6MWD by age, and Fig. 2 by height and weight, in females and in males separately. The adjusted mean of 6MWD displayed a different trend by age according to the height of subjects, in males (Fig. 3): taller subjects showed greater average 6MWD but with an earlier and faster decline with respect to shorter subjects.

Table 5 gives the sex-specific equations for prediction of 6MWD. The R squared values of these models were 0.2381 in women and 0.1592 in men. The lower limit of the normal range (LLN) for a subject may be calculated by subtracting 122 or 96 m from the 6MWD value for men and women, respectively.

**Table 5:** Reference equations for 6MWD (m) in young, middle-aged and elderly healthy subjects

Predicted 6MWD^a^	LLN^b^
Females	
8.10*age + 1.61*height−0.99*weight + 22.58*active−0.10*age^2^ + 222.55	Subtract 96 m^b^
Males	
26.80*age + 8.46*height−0.45*weight−2.54*active−0.06*age^2^−0.13*age*height−890.18	Subtract 122 m^b^

Age is expressed in years, height is measured in centimeters (cm) and weight in Kilograms (Kg). Active is a dichotomous variable: its value is 1 when the subject is physically active, it is 0 otherwise; subjects who performed physical activity with a frequency of ‘‘2–3 times a week’’ or more and with a duration of ‘‘about 1 h a week’’ or more were classified as ‘‘active’’, the remaining subjects as ‘‘not active’’

^a^The reference equations for females and males presented in this table were calculated considering that the coefficient estimates were obtained with centered variables

^b^LLN = predicted mean—1.645 * RSD, where RSD is the residual standard deviation of the estimated regression and −1.645 is the z-score used to detect if the measured value of the 6MWD is lower than the 95% of the subjects with the same age, height, weight and level of physical activity

## Discussion

To our knowledge, this is one of the few studies evaluating 6MWD in a large general population cohort of Caucasian healthy subjects, with one of the widest age-range so far considered. We confirmed the importance of sex, age, height, weight as determinants of the distance walked, and also showed that physical activity influences the results of the test. Furthermore, we found evidence that the relationship between age and the distance walked is not linear but is better represented adding a quadratic term, and an interaction between age and height is present in males. Finally, we propose new standard equations as reference for the clinical practice.

In agreement with the previous studies, the average 6MWD was significantly different between sexes, with men walking a greater distance than women. This could be due to the fact that men are taller, have a greater absolute muscle strength and mass, and may express a greater aerobic power. As a novelty with respect to previous studies, we found that the discrepancy between males and females is not only related to the metres covered, but it is also due to differences in the determinants of the distance walked. Weight negatively affects the distance travelled in females, but not in males; additionally, self-reported physical activity appears to be associated with 6MWD only in women; finally, an interaction between age and height was seen exclusively in men.

One of the factors affecting the 6MWD was age, a result that has been observed in almost all previous studies. The negative influence of age is evident from around 40 years of age and appears to be even more important as age increases, both in females and males. The progressive reduction in skeletal muscle mass that occurs with age [14], may justify these results. It should be noted that lean mass (a marker of skeletal muscle mass, especially when measured in the limbs) presents a different behaviour in the two sexes: in males it decreases from 50–60 years of age, in females tends to steadily increase, albeit slightly [28]; these trends do not correspond to the similar trajectories seen in our study in both sexes, indicating that other age-related factors, such as progressive loss of strength and increased prevalence of debilitating diseases, may play a role.

A taller height is associated with a longer pass, which may result in a longer distance travelled. Increased vital capacity associated with higher stature is likely to contribute to better performance of taller individuals. The interaction of height with age, first documented in the present study in males, is of interest. The subjects of shorter stature appear to achieve the best physical performance expressed by 6MWD between 55 and 60 years, followed by a slow decline in the distance covered. The taller subjects, on the other hand, have their best performance at a younger age, around 20–30 years, but then the functional decline is progressively faster until the performance tends to be worse than the shorter individuals after 65 years. The data in this study do not provide an explanation for these results or for the fact that the interaction is only present in males. However, it is likely that multiple factors such as differences in body composition, in the mechanics of walking, may explain these findings. Taking into account that physical performance is a key contributor to late life mobility and independence [29], the finding of a rapid functional decline in taller individuals has practical implications, since early detection and treatment of functional derangement may play a crucial role in delaying the onset of functional impairment and physical disability [30].

Overweight and obesity increase the workload for a certain amount of exercise, thus explaining the shorter distance travelled by women with higher body weight. There was no relationship between walking distance and weight in men, in agreement with the data reported by Zou et al. [11]. Although there is no definitive explanation for this, we speculate that this may be due to the different body composition. Particularly, the lower lean mass in women (which is a marker of muscle mass [28]), would make them less tolerant of the weight-related increase in load. An alternative hypothesis is that gender differences in body fat distribution may lead to decreased gait efficiency in females [31]. The significant contribution of weight is in partial agreement with the findings of Enright [14], Trooster [8], Casanova [12], Zou [11], Oliveira [13], suggesting that when 6MWDs are reported in future studies, they should also be corrected for weight.

Physiology studies have shown that physical exercise is positively related to muscle strength, whereas a sedentary lifestyle alter muscle mass, metabolism and function [32]. Moreover, previous work has shown a relationship between physical activity and physical performance [33, 34]. Particularly, Hall et al. [35] reported that poor performance during the 6-min walk was significantly associated with reduced physical activity in a study of 775 individuals aged 30–90 years. Our finding of effect of self-reported physical activity on the 6MWD is consistent with these results [35] and those by Zou et al. [11], but it is in contrast with other studies that failed to demonstrate this association [7, 9, 12].

The same variables were considered for consistency in the reference equations of 6MWD for both sexes, even if weight and physical activity were not statistically significant in males. It is possible that we failed to consider other potential sources of variability. One source of variance may be the different attitudes and the mood of the participants [36, 37]. The speed of habitual walking and other aspects related to the motivation of the subject and/or the assessor [12] may also have influenced the distance covered. Furthermore, it is possible that the anthropometric variables measured may not include all the anthropometric information necessary to explain variability in the 6MWD. For example, lean body mass, a parameter known to predict exercise capacity in healthy subjects, has not been measured [38].

Several published studies have provided 6MWD prediction equations for healthy adults [8, 11–14]. These studies examined a wide range of populations and methodologies, making it difficult to compare our results with those of other studies. We compared our results with those of Enright and Sherrill [14], a study similar to our own as conducted in a large general population sample, although with a smaller age range of participants. If we consider only subjects over 40 years of age, Enright and Sherrill equations underestimate the 6MWD in our sample by 6 m in females and overestimate it by 13 m in males.

The strength of the study is that participants were a large sample from the general population, represented all age groups and were well balanced by sex.

We need to acknowledge the limits of the study. The reference curves, although spanning a wide age range, cannot be used in non-Caucasian subjects and in subjects over 78 and 76 years of age for males and females, respectively. However, this range is the age at which the vast majority of the patients with cardiopulmonary pathologies manifest their illnesses. We did not measured the ΔHR (the difference between hearth rate at the end of the test and HR at rest), a parameter representing the level of effort the subject expends during the test [11] and that has been reported to be positively associated to the 6MWD [13].

The final regression equations explained 24% and 16% of the variance in the 6MWD for females and males, respectively. These values are not particularly high, but the use of R squared as a measure of goodness of fit is highly controversial. In particular, Hanushek and Jackson [39] advise extreme caution when comparing R squared from different samples. With the aim of evaluating the fitting of our models to data, we considered it more important to check whether the models were misspecified through the Ramsey test and to assess whether the assumptions of linear regression (normally distributed errors with constant variance) had been met.

Finally, we recognise that higher values may be expected from individuals who have previously performed a 6-min walk test as a result of the learning effect when the test is performed on two consecutive occasions [40]. However, this effect is not crucial when determining a cross-sectional relationship or when utilizing the results as a baseline predictor of future event.

## Conclusion

The present study was the first to describe the 6MWD in a large population sample of young, middle aged and elderly healthy Caucasian subjects and to formulate a predictive equation, thus contributing to improve the evaluation of European patients with diseases affecting their functional capacity. Age, sex, height, weight and physical activity with age-to-weight interaction in males, were the predictors of the 6MWD.

## Data Availability

The deidentified participant data are available upon reasonable request. The contact for this request is the corresponding author, and the request will be forwarded to the Steering Committee of the GEIRD study that will evaluate the request and decide whether to approve it.

## Abbreviations

6MWD: Six-minute walking distance
6MWT: Six-minute walking test
BMI: Body mass index
GEIRD: Gene Environment Interaction in Respiratory Diseases
LLN: Lower limit of normal
LRT: Likelihood ratio test
